# Rapid activation of ARF6 after RAF inhibition augments BRAFV600E and promotes therapy resistance

**DOI:** 10.21203/rs.3.rs-7133814/v1

**Published:** 2025-08-27

**Authors:** Allie Grossmann, Junhua Wang, Yinshen Wee, Thomas Jacob, Aaron Rogers, Lise Sorensen, Deja Brooks, Prachi Gupta, Joshua Tay, Emily Wilson, Tong Liu, Eric Smith, Vashisht YN, Michael Davies, Martin McMahon, Sheri Holmen, Robert Judson-Torres, Roger Wolff

**Affiliations:** Providence Cancer Institute of Oregon; University of Utah; Taipei Medical University; Providence Cancer Institute of ORegon; University of Utah; University of Utah; Providence Cancer Institute of Oregon; Providence Cancer Institute of Oregon; University of Utah; University of Utah, Huntsman Cancer Institute; University of Utah, Huntsman Cancer Institute; University of Utah, Huntsman Cancer Institute; The University of Texas MD Anderson Cancer Center; MD Anderson Cancer Center; Huntsman Cancer Institute; Huntsman Cancer Institute; Huntsman Cancer Institute; University of Utah

## Abstract

The intrinsic ability of cancer cells to evade death underpins tumorigenesis, progression, metastasis and the survival of drug-tolerant persister (DTP) cells. Herein, we discovered that when activated, the small GTPase ARF6 plays a central role in tumor survival by facilitating expression of the BRAF^V600E^ oncoprotein. Tumor-specific *Arf6* deletion caused a significant reduction in BRAF^V600E^ protein and MAPK signaling and prevented rapid tumor progression. In the context of targeted therapy, BRAF inhibition induced swift activation of ARF6, driving a positive feedback loop that restored MAPK-driven anti-apoptotic signaling, facilitated DTP cell survival during the early phases of treatment and contributed to drug-tolerant growth. In patient-derived melanoma cells with innate or clinically acquired resistance to MAPK inhibitors, ARF6 inhibition enhanced sensitivity to combined BRAF + MEK inhibition. Collectively, these findings elucidate an ARF6-dependent mechanism of BRAF oncoprotein synthesis that may be exploited in BRAF^V600E^ driven cancers as a therapeutic vulnerability.

## Introduction

Avoiding cell death is fundamental to the progressive acquisition of hallmark cancer behaviors^1^, and to the survival of drug tolerant persister (DTP) cells that give rise to therapy resistance^2^. Proposed origins of DTP cells include clonal selection of pre-existing drug-resistant cells, and drug-induction of a reversible DTP state that enables the outgrowth of fixed resistance^3, 4^. The molecular mechanisms of DTP cell emergence are complex and incompletely understood.

Mitogen activated protein kinase (MAPK) signaling is hyperactivated in many cancer types and directly opposes cell death by inactivating pro-apoptotic proteins of the intrinsic pathway^5^. In cutaneous melanoma, where most cases harbor somatic mutations in *BRAF, NRAS* or *NF1* that cause aberrant MAPK signaling^6^, resistance to MAPK targeted therapy remains a significant clinical challenge^7^.

We detected abnormally elevated levels of ARF6-GTP, the active form of the small GTPase ARF6, in melanoma^8^. ARF6 activation in melanoma can occur through extracellular signals such as HGF^9, 10^, WNT5a^11^ and Interferon-g^12^; or by altered expression of guanine exchange factors (GEFs) or GTPase activating proteins (GAPs)^8, 12, 13^. HGF and WNT5a signaling also mediate resistance to MAPK targeted therapy in melanoma^14–17^. We showed that ARF6-GTP promotes tumor development, progression, and acceleration of metastasis in murine models of BRAF^V600E^ melanoma^8, 11, 12^. Functionally, ARF6 activation enhances tumor cell invasion and adaptive immune suppression^8, 9, 11, 12^. Hence, ARF6 mediates the acquisition of at least two hallmark malignant behaviors^1^. Whether ARF6 is involved in DTP cell biology is unknown.

ARF6 is a ubiquitously expressed protein critical for endomembrane trafficking and actin cytoskeleton remodeling^18^ and has diverse physiologic roles across multiple organ systems^19–30^. To expand our understanding of ARF6 function in cancer, we interrogated proteomic alterations induced by ARF6 activation and discovered that ARF6 dynamically regulates expression of the BRAF oncoprotein in melanoma and other cancer types, impacting tumor cell survival, including during MAPK targeted therapy.

## RESULTS

### ARF6 augments a dynamic pool of oncogenic BRAF protein

**Figure 1:**
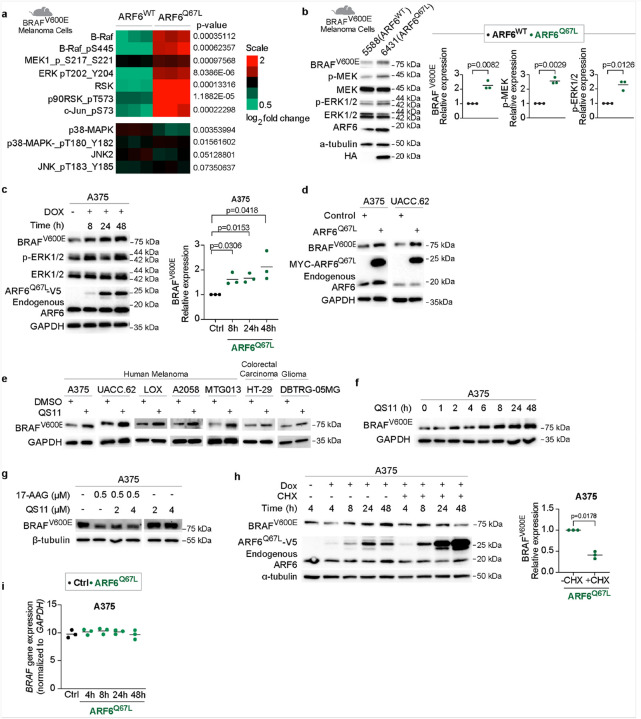
ARF6 is sufficient to control oncogenic BRAF protein levels through protein translation. **a**, Relative amount of MAPK signaling proteins in tumor cells derived from *Braf*^*V600E*^*/ Cdkn2a*^*f/f*^ mice detected by Reverse Phase Protein Array, two-tailed t-test. n= 3 replicates per cell line. **b-h**, Western Blot for indicated proteins. **b**, murine melanoma cells derived from *Braf*^*V600E*^*/ Cdkn2a*^*f/f*^ mice. n=3 biological independent experiments **c**, dox-inducible ectopic expressed ARF6^Q67L^. n=3 biological independent experiments **d**, Western blot for indicated proteins in UACC.62 and A375 cells with or without adenoviral-mediated ectopic expression of ARF6^Q67L^, control= empty vector. **e**, 4μM QS11 for 48h in A2058, HT-29, and DBTRG-05MG. 2μM QS11 for 24h other cell lines **f**, 2μM QS11. **g**, 17-AAG and QS11 for 24h in A375 with doxycycline-inducible ectopic expressed ARF6^Q67L^. **h**, 20μg/ml cycloheximide (CHX) in A375 with doxycycline-inducible ectopic expressed ARF6^Q67L^. BRAF^V600E^ protein quantification at 48h. n=3 biological independent experiments. i, Quantitative RT-PCR for *BRAF* mRNA in A375 with doxycycline-inducible ectopic expressed ARF6^Q67L^, n=3 biological independent experiments. **b, c, h**, Two-tailed ratio paired t-test.

In early passage murine melanoma cell lines with homozygous *BRAF*^*V600E*^ mutation, derived from our genetically engineered murine melanoma models^8, 12^, proteomic analysis showed higher levels of BRAF^V600E^ protein, and increased phosphorylated MEK1, ERK, RSK and Jun, in cells expressing constitutively active ARF6-GTP (ARF6^Q67L^) compared to ARF6^WT^ (Fig. 1a). In contrast, p38 MAPK-JNK signaling was unaltered (Fig. 1a). ARF6-GTP-induced BRAF^V600E^ expression was confirmed by Western blot (Fig. 1b). These findings align with our previously published genomic data from this tumor model showing upregulation of genes in the MAPK cascade in bulk tumor transcriptomes^8^. Based on these findings, we hypothesized that ARF6 controls MAPK signaling by regulating oncogenic BRAF expression. In pursuit of this, we interrogated human melanoma cells and found that doxycycline-induced, ectopically expressed ARF6-GTP, in the form of ARF6^Q67L^ (**Fig. S1a**), or adenoviral delivered ARF6^Q67L^, augmented endogenous BRAF^V600E^ expression in human melanoma cells (Fig. 1c–d). Consistent with genetic activation of ARF6, pharmacological activation of ARF6 with QS11 (**Fig. S1b**), an inhibitor of ARF GTPase Activating Protein 1^31^, increased BRAF^V600E^ protein expression in human melanoma, colorectal carcinoma and glioma cell lines (Fig. 1e, **S1c**). BRAF^V600E^ protein levels rose quickly after treatment with the ARF6 agonist QS11, as early as two hours, and continued to accumulate over 48 hours (Fig. 1f). These data demonstrate that sustained ARF6 activation is sufficient to acutely increase endogenous BRAF^V600E^ protein.

The BRAF^V600E^ oncoprotein is stabilized by the chaperone protein HSP90^32, 33^, limiting proteasome-mediated degradation. Unlike HSP90, ARF6 prevents lysosome mediated degradation of proteins through endosomal recycling^12, 34, 35^. Thus, we asked if BRAF^V600E^ might be degraded by the lysosome. Blocking lysosomal degradation by Bafilomycin A1 failed to increase BRAF^V600E^ protein (**Fig. S1d**). Thus, it is unlikely that ARF6 regulates oncogenic BRAF expression through endolysosomal trafficking. ARF6-GTP did not alter HSP90 protein expression (**Fig. S1e**). Nevertheless, the ARF6-regulated pool of BRAF^V600E^ was HSP90 dependent because inhibition of HSP90 with 17-AAG prevented accumulation of BRAF^V600E^ protein after QS11 treatment (Fig. 1g).

Surprisingly, ARF6 mediated BRAF^V600E^ expression was dependent on protein translation. Specifically, inhibition of protein translation with cycloheximide prevented the accumulation of BRAF^V600E^ protein upon ARF6 activation (Fig. 1h). Activation of ARF6 failed to alter *BRAF* mRNA levels in A375 melanoma cells, which harbor a homozygous *BRAF*^*V600E*^ mutation (Fig. 1i), demonstrating that ARF6-mediated upregulation of BRAF^V600E^ occurred without altering *BRAF* oncogene expression. These data confirm that ARF6 activation is sufficient to increase BRAF^V600E^ protein levels and suggest a previously unknown role for ARF6 in regulating protein translation.

### ARF6 is necessary for maintenance of the BRAF protein

**Figure 2:**
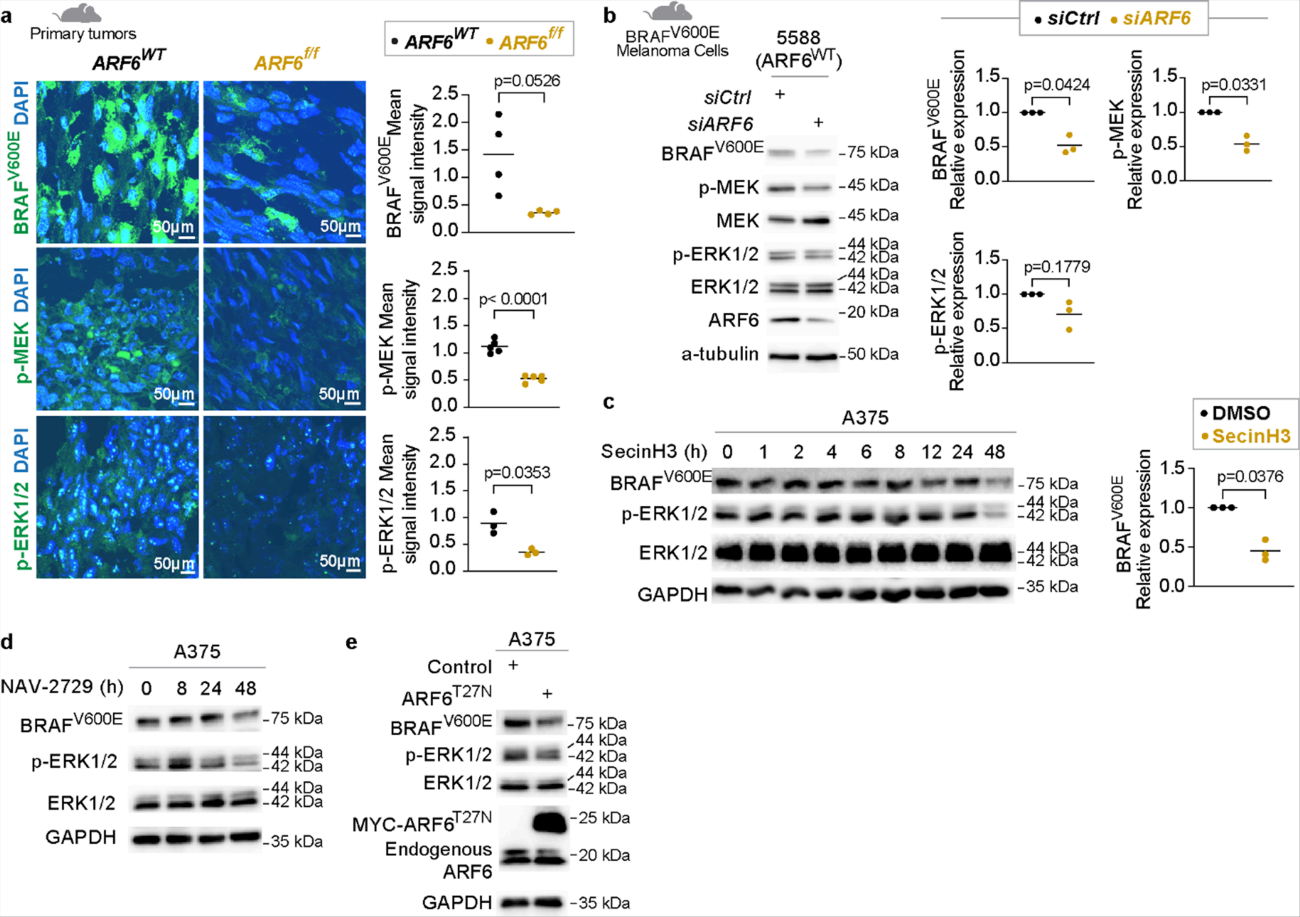
ARF6 is necessary to control oncogenic BRAF protein levels. **a**, Representative immunofluorescence images of cryo-embedded frozen tumor tissues from *Braf*^*V600E*^*/Cdkn2a*^*f/f*^ mice, 1200X magnification. **b-e**, Western blot for indicated proteins. b, murine melanoma cells derived from *Braf*^*V600E*^*/ Cdkn2a*^*f/f*^ mice, n=3 biological independent experiments. c, 10μM SecinH3, BRAF^V600E^ protein quantification at 48h, n=3 biological independent experiments. **d**, 5μM NAV-2729. **e**, A375 cells with or without adenoviral-mediated ectopic expression of ARF6^T27N^, control= empty vector. **a**, Two-tailed unpaired t-test. b, e, Two-tailed ratio paired t-test.

In contrast to ARF6 activation, deletion of *Arf6* in BRAF^V600E^ murine melanoma tumors^12^ reduced total BRAF^V600E^ levels and downstream phosphorylated MEK (p-MEK) and ERK (p-ERK), detected by immunofluorescence *in situ* (Fig. 2a, **S1f**). Consistently, silencing of *Arf6* downregulated BRAF^V600E^ and p-MEK detection in murine melanoma cells (Fig. 2b). To test whether inactivation of ARF6 (ARF6-GDP) could produce the same effect, we treated human melanoma cells with SecinH3, an ARF6 guanine exchange factor inhibitor that reduces ARF6-GTP levels^11, 36^ (**Fig. S1g**) and reduces spontaneous metastasis of human BRAF^V600E^ melanoma xenograft tumors^11^. In human melanoma cells, SecinH3 significantly reduced BRAF^V600E^ protein within 48 hours of treatment (Fig. 2c). NAV-2729, a direct inhibitor of ARF6 GTPase function^13^ (**Fig. S1h**), also reduced BRAF^V600E^ protein after 48 hours (Fig. 2d). Finally, ectopic expression of inactive ARF6 (ARF6-GDP), in the form of ARF6^T27N^, reduced BRAF^V600E^ protein (Fig. 2e), suggesting that ARF6 activation is necessary to maintain expression of endogenous BRAF^V600E^. Together these data demonstrate that ARF6 may be necessary to maintain steady state levels of the BRAF^V600E^ oncoprotein and suggest that targeted inhibition of ARF6 might be an alternative approach to reducing BRAF^V600E^ oncoprotein expression.

### ARF6-GTP promotes tumor survival by protecting against apoptosis

**Figure 3:**
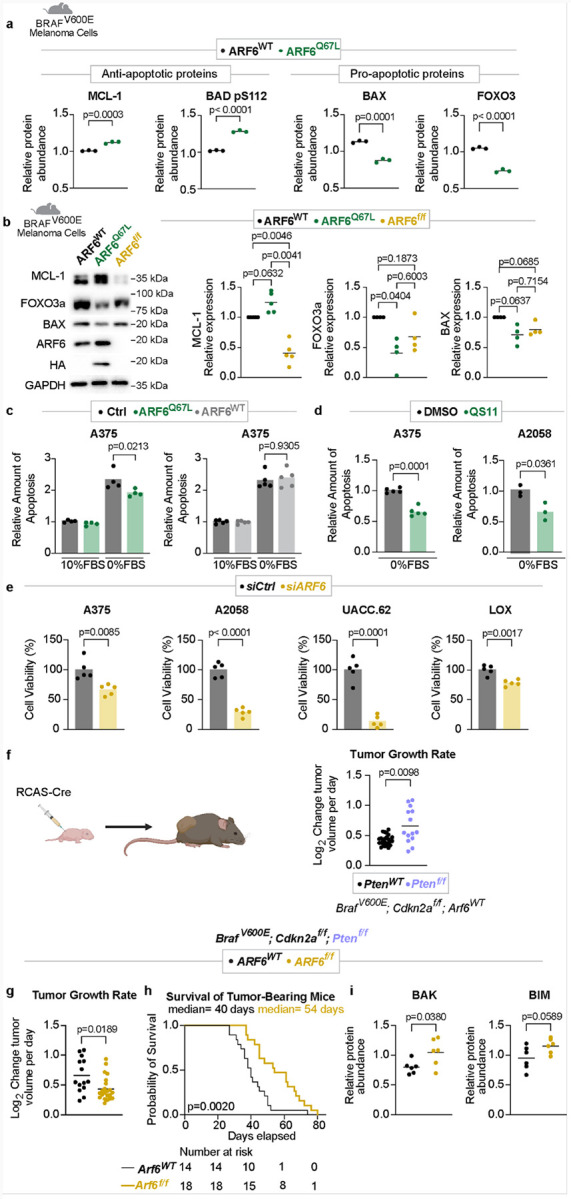
ARF6 promotes tumor survival and accelerated disease progression. **a**, Apoptotic protein profile of tumor cells derived from *Braf*^*V600E*^*/ Cdkn2a*^*f/f*^ mice detected by Reverse Phase Protein Array, two-tailed t-test. n= 3 replicates per cell line. **b**, Western Blot for indicated proteins in murine melanoma cells derived from *Braf*^*V600E*^*/ Cdkn2a*^*f/f*^ mice, n=3 biological independent experiments. Two-tailed ratio paired t-test. **c**, Apoptosis detection, measured at 48h, dox-inducible ectopic expressed ARF6^WT^ and ARF6^Q67L^ in A375 cells, n=4 replicates per condition, One-way ANOVA with multiple comparisons. **d**, Apoptosis detection, 4μM QS11, measured at 48h, n=5 for A375 and n=3 for A2058 replicates per condition, Two-tailed unpaired t-test. **e**, Cell viability detection, measured at 72h, n=5 replicates per condition. Two-tailed unpaired t-test. **f**, Rate of tumor growth measured from the time of initial detection in *Dct::TVA;Braf*^*V600E*^;*Cdkn2a*^*f/f*^ mice, n= 24 *Pten*^*WT*^, n=14 *Pten*^*f/f*^ mice, two-tailed t-test with Welch’s correction. **g**, Rate of tumor growth measured from the time of initial detection in *Dct::TVA;Braf*^*V600E*^;*Cdkn2a*^*f/f*^;*Pten*^*f/f*^ mice, *n* = 14 *Arf6*^*WT*^, *n* = 22 *Arf6*^*f/f*^ mice, two-tailed t-test with Welch’s correction. **h**, Survival of mice (before primary tumor reached 2 cm) after Cre injection (day 0) within 130 days, *n* = 14 *Arf6*^*WT*^, *n* = 18 *Arf6*^*f/f*^ mice, Log-rank (Mantle-Cox) test. Solid line withing data points = mean. **i**, Apoptotic protein profile of tumors from *Dct::TVA;Braf*^*V600E*^;*Cdkn2a*^*f/f*^;*Pten*^*f/f*^ mice (n=6 mice per group) detected by Reverse Phase Protein Array, two-tailed t-test.

Because MAPK signaling opposes the intrinsic apoptotic signaling pathway^5^, we reasoned that ARF6-mediated fluctuations in BRAF^V600E^protein might be linked to survival. Proteomic clues to ARF6-mediated survival were evident in murine melanoma cell lines cultured in full serum (Fig. 3a–b). Compared to ARF6^WT^, cells expressing ARF6^Q67L^ showed significantly increased levels of the anti-apoptotic protein MCL-1 and phosphorylation (inactivation) of BAD at residue S112 (pS112)^5^, as well as decreased levels of pro-apoptotic proteins BAX and FOXO3 (Fig. 3a). ARF6 dependent expression of MCL-1 and FOXO3 were confirmed by Western blot (Fig. 3b). ERK signaling has been reported to increase MCL-1^37^ and decrease FOXO3^38^ protein levels^5^. Thus, our data suggest that ARF6 activation might promote tumor cell survival through ERK-mediated anti-apoptotic signaling.

To test whether ARF6 activation could protect against apoptosis, we deployed a doxycycline-inducible system to express either ectopic ARF6^Q67L^ or ARF6^WT^ in human melanoma cells (**Fig.S1a, S2a**). Doxycycline alone did not alter viability of A375 parental cells, (**Fig. S2b**), while doxycycline-induced ARF6^Q67L^ significantly reduced apoptosis caused by serum withdrawal (Fig. 3c). In contrast, doxycycline-induced ectopic expression of ARF6^WT^ did not alter apoptosis caused by serum withdrawal (Fig. 3c), suggesting that the active form of ARF6 is required for the survival benefit. Consistent with ARF6^Q67L^, pharmacological activation of ARF6 with QS11 protected against apoptosis caused by serum starvation (Fig. 3d). QS11 alone failed to alter cell viability during steady-state conditions, when cells were cultured in full serum (**Fig. S2c**), indicating that the compound does not stimulate proliferation. Overall, these data demonstrate that ARF6 activation can protect against apoptosis during growth signal deprivation.

Given that ARF6 can regulate both PI3K-AKT^8^ and BRAF^V600E^ -MAPK signaling (Fig. 1) and apoptosis upon serum withdrawal (Fig. 3c–d), we asked if ARF6 supports the viability of BRAF-mutant human cancer cells grown in full serum. Consistent with this, *ARF6* silencing led to significantly reduced viability in multiple human melanoma cell lines (Fig. 3e). Similarly, treatment with NAV-2729, a direct inhibitor of ARF6 GTPase function^13^, reduced ARF6-GTP levels (**Fig. S1h**) and decreased cell viability in most of the human melanoma cells tested (**Fig. S2d**), although not as effectively as *ARF6* silencing (Fig. 3e). These data demonstrate that ARF6 can optimize survival during normal growth conditions.

### ARF6 is required for accelerated tumor progression caused by PTEN loss

In parallel with the MAPK pathway, survival signaling can also originate from the PI3K-AKT pathway^39^ and we previously reported that activation of ARF6 enhanced PI3K expression and PI3K-AKT signaling^8^. *PTEN* loss of function mutations activate the PI3K-AKT pathway, are frequently detected in cutaneous melanoma^6^, cooperate with mutant BRAF or NRAS to drive melanomagenesis^40, 41^, and accelerate primary tumor growth in genetically engineered *Dct::TVA, Braf*^*V600E*^; *Cdkn2a*^*flox/flox*^ murine melanoma models induced in epidermal melanocytes of the ear pinnae^42^. Like pinnae tumors, deletion of *Pten* dramatically accelerated the growth of BRAF^V600E^ melanoma induced in the flank (Fig. 3f). To test the necessity of ARF6 in this highly aggressive model, we crossed *Arf6*^*flox/flox*^ (*Arf6*^*f/f*^) mice with the *Dct::TVA, Braf*^*V600E*^; *Cdkn2a*^*f/f*^; *Pten*^*f/f*^ mice. In this model, tumor-specific loss of *Arf6* significantly reduced tumor growth to a level equivalent to *Pten*^*WT*^ tumors (measured from the time of tumor formation, Fig. 3g), and prolonged overall survival despite the absence of PTEN (Fig. 3h). Unlike *Pten*^*WT*^ mice^12^, loss of ARF6 did not reduce overall tumor incidence in *Pten*^*f/f*^ mice (**Fig. S2e**), demonstrating that loss of PTEN is sufficient to overcome the weakened tumor initiation phenotype we previously observed with *Arf6* knockout. Nevertheless, loss of ARF6 significantly delayed tumor onset in *Pten*^*f/f*^ mice (**Fig. S2e**). Consistent with the *Pten*^*WT*^ tumor cell lines (Fig. 3b), tumors from *Pten*^*f/f*^; *Arf6*^*f/f*^ mice showed increased levels of pro-apoptotic proteins BAK and BIM (Fig. 3i), suggesting enhanced apoptosis signaling in the absence of ARF6. Given that *Arf6* deletion prevented primary tumor acceleration caused by PTEN loss (Fig. 3f–g), there is a component of ARF6-dependent survival that is necessary for, and/or functions independently of the PI3K pathway. Indeed, ARF6-dependent survival may also originate from rheostatic control of BRAF^V600E^ expression (Figs. 1–2) and downstream, MAPK-mediated anti-apoptotic signaling.

### ARF6 is activated by RAF inhibition, protects against MAPK inhibitor-induced apoptosis, and potentiates resistance to MAPK inhibition

**Figure 4:**
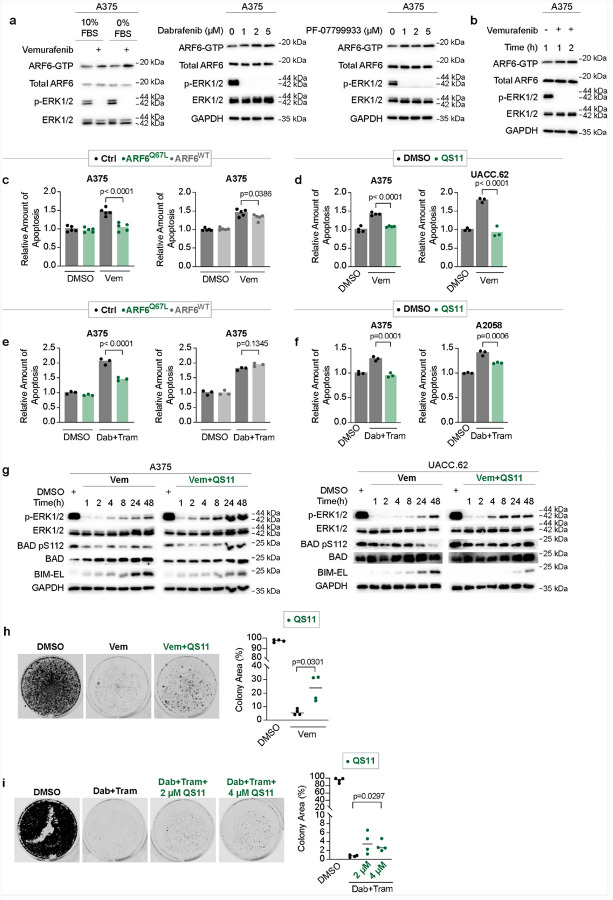
ARF6 activation protects against MAPKi-induced apoptosis and promotes the development of MAPKi-resistance cells. **a-b**, Total ARF6 and ARF6-GTP pulldown in A375, 5μM vemurafenib for 4h, Dabrafenib treatment for 4h,PF-07799933 treatment for 2h in 0%FBS media. **b**, 5μM vemurafenib. **c-f**, Apoptosis detection. **c**, 1μM Vemurafenib, dox-inducible ectopic expressed ARF6^WT^ and ARF6^Q67L^ in A375, apoptosis measured at 48h, Ctrl= no doxycycline, n=5 replicates per condition. **d**, 1μM Vemurafenib, 4μM QS11 for A375, n=4 replicates per condition, apoptosis measured at 48h, 2μM Vemurafenib, 4μM QS11 for UACC.62, n=3 replicates per condition, apoptosis measured at 24h. **e**, 1.25μM Dabrafenib, 0.0625μM Trametinib, doxinducible ectopic expressed ARF6^WT^ and ARF6^Q67L^ in A375, apoptosis measured at 48h, Ctrl= no doxycycline, n=3 replicates per condition. **f**, 1.25μM Dabrafenib, 0.0625μM Trametinib, 4μM QS11, apoptosis measured at 48h, n=3 replicates per condition. **g**, Western Blot for indicated proteins. 1μM Vemurafenib, 4μM QS11 in A375. 2μM Vemurafenib, 4μM QS11 in UACC.62. **h-i**, Colony outgrowth assay in A375. **h**, 1μM Vemurafenib, 4μM QS11, for 30 days. **i**, 250nM Dabrafenib, 12.5nM Trametinib, 2μM QS11, 4μM QS11, for 30 days. **c-f**, One-way ANOVA with multiple comparisons. **h-i**, Two-tailed unpaired t-test. n=4 biological independent experiments.

Because ARF6 can regulate BRAF^V600E^ protein expression (Figs. 1–2), we asked if BRAF inhibition alters ARF6 activation. Remarkably, class I BRAF inhibitors, vemurafenib or dabrafenib, increased ARF6-GTP levels (Fig. 4a). This occurred both in the presence and absence of serum and is reproducible in independent BRAF^V600E^ cell lines (Fig. 4a and **S3a**). Notably, the pan-mutant BRAF inhibitor PF-07799933, which inhibits BRAF mutant monomers and dimers and has antitumor activity in treatment refractory patients^43^, also increased ARF6-GTP levels in human melanoma (Fig. 4a). Importantly, ARF6 activation occurred rapidly after BRAF inhibition, as early as one hour (Fig. 4a–b), suggesting that ARF6 activation functions in an acute adaptive response pathway to BRAF-targeted therapy.

Because ARF6 was rapidly activated upon RAF inhibition and ARF6-GTP promoted survival upon serum withdrawal (Figs. 4a–b, 3c–d), we asked whether ARF6 activation can facilitate survival during MAPK inhibitor (MAPKi) treatment. Indeed, genetic activation of ARF6 dramatically reduced apoptosis after 48 hours of vemurafenib (Fig. 4c), whereas silencing of *Arf6* significantly increased apoptosis induced by vemurafenib (**Fig S3b**), consistent with a role for ARF6 in early tumor cell survival during targeted therapy. Overexpression of wildtype ARF6 also decreased vemurafenib-induced apoptosis, but to a lesser extent than ARF6^Q67L^ (Fig. 4c). Similar to ARF^Q67L^, pharmacological activation of ARF6 with QS11 almost completely abrogated vemurafenib induced apoptosis (Fig. 4d).

Combination RAF + MEK inhibition is the preferred choice of MAPKi therapy in BRAF^V600E^ melanoma patients, due to superior clinical outcomes compared to single agent RAF inhibition^44^. Thus, we interrogated ARF6 in this context. A375 melanoma cells are highly sensitive to both single-agent RAF inhibition and combination RAF + MEK inhibition in short-term cultures (**Fig S3c-d**). In contrast, A2058 melanoma cells are resistant to vemurafenib (**Fig S3c**), possibly due to a *MAP2K1* P124S mutation^45^, but remain sensitive to the combination of dabrafenib + trametinib (Dab + Tram) (**Fig S3d**). Importantly, genetic or pharmacologic activation of ARF6 reduced Dab + Tram sensitivity in these cell lines by significantly reducing apoptosis (Fig. 4e–f). These combined data suggest that the consequence of ARF6 activation upon BRAF inhibition (Fig. 4a–b) might be the emergence of resistance.

Because ARF6 activation can fortify BRAF^V600E^ protein (Fig. 1), we reasoned that ARF6 might facilitate recovery of MAPK signaling after RAF inhibition. Indeed, ARF6 activation by QS11 resulted in a markedly faster recovery of phosphorylated ERK (pERK) after vemurafenib treatment (Fig. 4g and **Fig. S3e**). Additional evidence that ARF6-GTP boosted MAPK recovery manifested in ERK-mediated inhibition of the apoptotic proteins BAD and BIM^5^. Unlike the control, QS11 significantly recovered ERK-mediated phosphorylation (inhibition) of BAD 24–48 hours after vemurafenib (Fig. 4g and **S3e**). Furthermore, downregulation of BIM was more pronounced with QS11 (Fig. 4g and **S3e**). These findings demonstrate that ARF6 activation can potentiate MAPK reactivation and anti-apoptotic signaling after BRAF inhibition.

To test if ARF6-GTP promotes the emergence of DTP cells, leading to therapy resistance, we quantified colony formation during vemurafenib (Fig. 4h) or Dab + Tram treatment (Fig. 4i). Activation of ARF6 with QS11 significantly increased drug-resistant colony formation in both conditions (Fig. 4h–i, **S3f-g**). Hence, our overall data supports that ARF6 is activated in the early phases of adaptive resistance, acutely responding to diminished MAPK signaling, and facilitating the survival of drug-tolerant persister cells in melanoma.

### ARF6 inhibition sensitizes patient-derived, MAPK inhibitor-resistant melanoma cells

**Figure 5:**
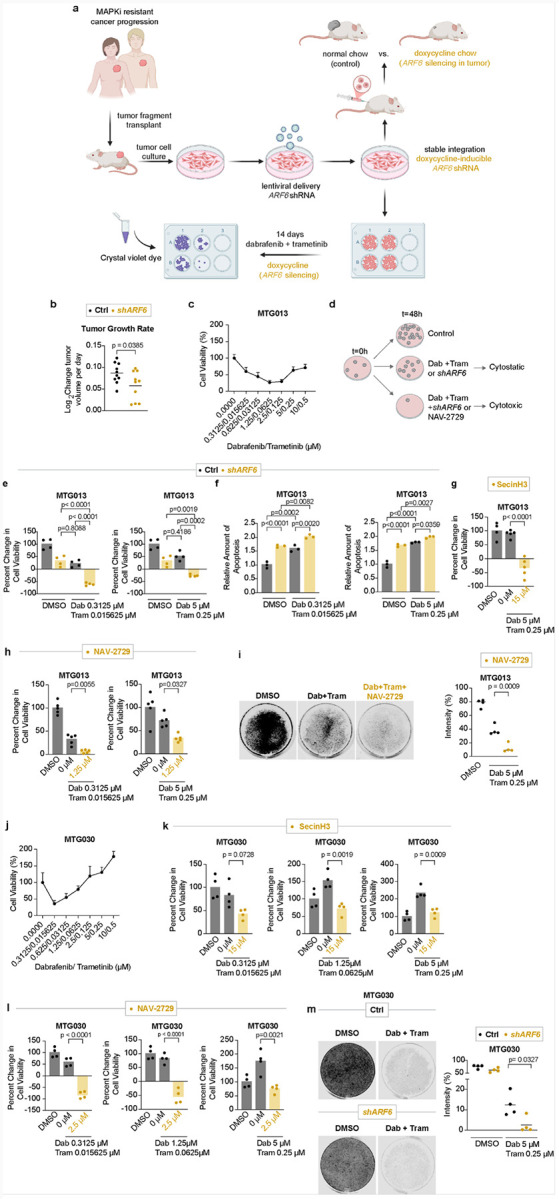
ARF6 inhibition sensitizes MAPKi-resistant cells. **a**, Schematics of *in vivo* and *in vitro* experiments with patient-derived xenograft cell lines. **b**,Rate of tumor growth measurements started six days after initial engraftment of MTG013 cells [stably transduced with doxycycline-induced short hairpin RNA (shRNA) for *ARF6*] in NRG mice, n=10 controls, n=10 fed doxycycline chow (*shARF6*), two-tailed t-test with Welch’s correction. **c, e, g, h, k, l**, Cell viability detection measured at 48 hours patient-derived cell lines (see Supplementary Table 1). **c**, Dose response to Dabrafenib plus Trametinib (Dab+Tram) in MTG013, n= 5 replicates per condition. **d**, Schematic of cell viability assay. **e-f**, Doxycycline-induced *shARF6*. **e**, n=4 replicates per condition. **f**, Apoptosis detection. n= 3 replicates per condition. **g, h, i, k, l**, pharmacologic inhibition of ARF6. **g**, n= 5 replicates per condition. **h**, n= 5 replicates per condition. **j**,Dose response of Dab+Tram in MTG030. n= 5 replicates per condition. **k**, n=4 replicates per condition. l, n=4 replicates per condition. **i, m**, Colony outgrowth assay in MTG013 and MTG030 for 14 days. **i**, MTG013 treated with 5 μM Dabrafenib and 0.25μM Trametinib and/or 1.25 μM NAV-2729. n=4 biological independent experiments. **m**, MTG030 cells treated with 5 μM Dabrafenib and 0.25μM Trametinib, Ctrl= no doxycycline. n=4 biological independent experiments. **e, f, g, h, j, k, l**, One-way ANOVA with multiple comparisons. **i, m**, Two-tailed unpaired t-test.

Because ARF6 activation significantly reduced tumor cell death after MAPKi (Fig. 4c–f), we asked whether inhibition of ARF6 could sensitize melanoma to clinically acquired or innate MAPKi resistance. For this, we pivoted to early-passage, patient-derived xenograft (PDX) melanoma cell lines (**Table S1**, Fig. 5a). We recently reported that the *MET* gene is amplified in MTG013/CM013 PDX cells^46^, which may explain the patient’s history of disease progression through vemurafenib treatment because HGF-MET signaling is a common mechanism of reactivation of MAPK signaling after RAFi^14^. Similar to the patient’s clinical outcome (progression through vemurafenib), MTG013 PDXs are resistant to high dose Dab + Tram^47^. We transduced these PDX cells with a doxycycline-inducible shRNA construct to conditionally knockdown *ARF6* expression after subcutaneous injection into immunodeficient NRG mice, or during *in vitro* colony forming assays (Fig. 5a). Doxycycline-induced knockdown of ARF6 significantly reduced tumor growth *in vivo* (Fig. 5b), demonstrating that ARF6 has a role in tumor progression that is independent of the ARF6-mediated adaptive immune suppression we observed in immunocompetent mice^12^. *In vitro*, MTG013 cells were increasingly resistant to rising concentrations of Dab + Tram (Fig. 5c), likely a result of progressive relief of an ERK negative feedback loop^5^ and reactivation of MAPK signaling^48^. From these Dab + Tram dose responses, we chose a low and a high dose Dab + Tram regimen to test in combination with knockdown (Fig. 5d–f) or pharmacologic inhibition of ARF6 (Fig. 5g, g, h). Change in viability was measured over 48 hours of treatment. By itself, silencing *ARF6* caused incomplete but significant loss of viability similar to Dab + Tram (Fig. 5e). Thus, inhibition of MAPK or ARF6 were equally cytostatic, but cell viability persisted above the baseline viability at time zero, indicating a low level of tumor cell survival (illustrated in Fig. 5d). Importantly, silencing of ARF6 resensitized MTG013 cells to Dab + Tram (Fig. 5e). Specifically, when *ARF6* knockdown was combined with Dab + Tram, there was a pronounced cytotoxic effect, where cell viability after 48 hours of treatment was less than time zero, and we observed this trend with both low and high combination doses of Dab + Tram (Fig. 5e). Consistently, silencing of ARF6 increased apoptosis induced by Dab + Tram (Fig. 5f). Like genetic depletion of ARF6, prevention of ARF6 activation with the ARF6 GEF inhibitor SecinH3^36^ (Fig. 5g), or direct inhibition of ARF6 with NAV-2729^13^ (Fig. 5h), decreased viability after Dab + Tram. NAV-2729 also significantly improved sensitivity to Dab + Tram during a 14- day colony outgrowth assay (Fig. 5i). Overall, the concordance between these orthogonal methods of ARF6 inhibition demonstrates reproducible efficacy in reversing clinically acquired MAPK inhibitor resistance.

Unlike MTG013, MTG030 cells have an increased copy number of *MAP2K1* (**Table S1**), which encodes for the BRAF substrate and effector protein MEK1. In addition, *HRAS* is amplified. These genetic changes may explain why these PDX melanoma cells were tolerant of Dab + Tram (Fig. 5j). In fact, intermediate to high doses of Dab + Tram enhanced tumor cell viability/growth in the first 48 hours of treatment (Fig. 5k, middle and right panels), and these cells appeared to be more resistant to MAPKi than MTG013 (Fig. 5c). The ARF6 GEF inhibitor, SecinH3, prevented the immediate burst in viability after Dab + Tram (Fig. 5k). Direct inhibition of ARF6 with NAV-2729 was cytotoxic when combined with low to intermediate doses of Dab + Tram (Fig. 5l, left and middle panels). Similar to SecinH3, NAV-2729 prevented the burst of enhanced viability that occurred with high dose Dab + Tram (Fig. 5l, right panel). With longer treatments (14 days), Dab + Tram reduced tumor colony formation, however, a low level of resistant tumor colonies persisted (Fig. 5m), and this was significantly diminished by knockdown of *ARF6* (Fig. 5m, **S3h**). Hence, these data suggest that targeting ARF6 may render melanomas with resistance mutations more vulnerable to MAPK inhibitors.

## DISCUSSION

**Figure 6:**
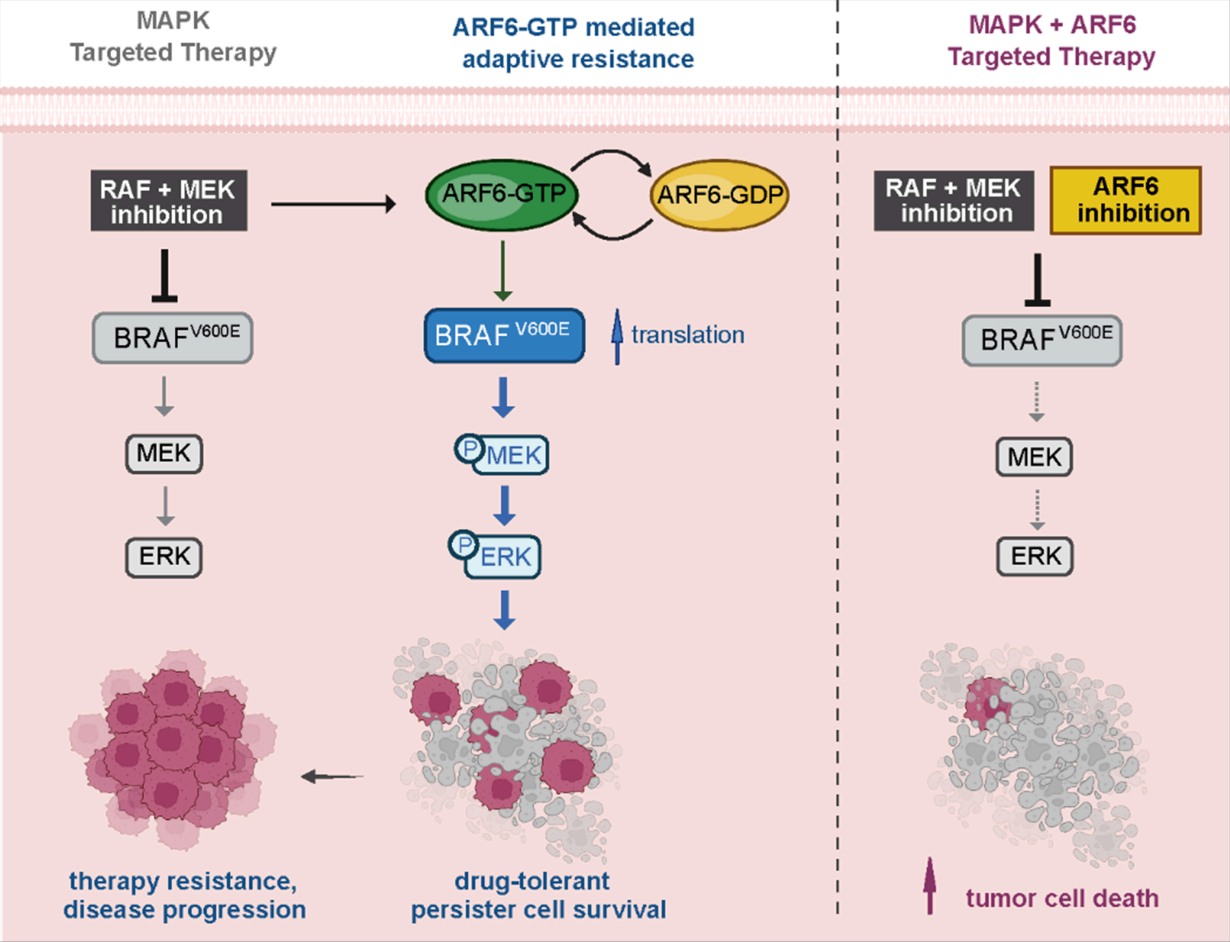
Proposed model of ARF6-dependent drug tolerant persister cell survival. Pharmacologic inhibition of BRAF^V600E^ induces ARF6 activation, triggering an adaptive stress response pathway that fortifies BRAF oncoprotein synthesis, reactivation of the MAPK pathway and DTP cell survival. Combined inhibition of ARF6 and MAPK signaling limits drug tolerance and enhances tumor cell death.

We have shown that the small GTPase ARF6 helps maintain BRAF^V600E^ protein expression through a post-transcriptional regulatory mechanism that stimulates BRAF^V600E^ translation. Without ARF6-GTP, BRAF^V600E^ protein levels gradually decline. Notably, ATP-competitive kinase inhibitors such as vemurafenib can reduce BRAF^V600E^ protein levels by preventing the HSP90 co-chaperone protein CDC37 from binding BRAF^49^. In this context, our findings suggest that cancer cells activate ARF6 in a positive feedback loop to maintain BRAF^V600E^ protein expression during kinase inhibition. Understanding how protein translation is deregulated in disease is important for the development of effective treatment approaches^50^. Messenger RNA translation occurs in cyclical bursts in mammalian cells^51^. Thus, a dynamic cycle of activation - deactivation of ARF6 might help stimulate pulsatile surges in BRAF^V600E^ synthesis to maintain steady-state levels, particularly when BRAF inhibitors are present and trigger ARF6 activation. While more work is needed to understand the mechanistic underpinnings and the potential extent of ARF6 regulation of protein expression, our data suggests that sustained inhibition of ARF6 can diminish BRAF^V600E^ levels and help overcome established resistance to MAPK targeted therapy (Fig. 6).

Our findings suggest that targeting ARF6 inhibits a stress-adaption pathway that gives rise to DTP cells. ARF6 mediated survival both during growth factor scarcity and MAPK targeted therapy. In the latter scenario, ARF6 was rapidly activated after initiation of RAFi treatment and mediated adaptive recovery of MAPK signaling. ARF6-GTP facilitated survival during the first few days of MAPKi therapy and enabled the eventual emergence of drug-resistant growth. Overall, our data support the hypothesis that DTP cells can be drug-induced^3, 4^ and provide mechanistic insights into how this phenomenon might occur in BRAF mutant cancers.

Our findings not only help explain how BRAF-mutant melanoma survives the acute phases of MAPK inhibition, they also highlight an emerging theme of pro-invasive small GTPases that link mechanisms of tumorigenesis to drug resistance. Like ARF6, the small GTPase RAC1 facilitates invasion^52^, tumorigenesis^52, 53^ and resistance to MAPK targeted therapy^53, 54^. Recently, RAC1 was shown to be activated by MEK inhibition^52^. Interestingly, RAC1 was activated in human melanoma cells between 8–16 hours after the initiation of treatment with trametinib. In contrast, ARF6 was activated within 1–2 hours of BRAF inhibition (Fig. 4a–b). The difference in kinetics could be due to the choice of MAPKi (MEK vs. BRAF), the use of different cell lines, or possibly due to distinct upstream mechanisms that result in serial activation of these small GTPases; ARF6 followed by RAC1. Unlike ARF6, however, activation of RAC1 was not reported to signal through the MAPK pathway^53^. Like RAC1 and ARF6, RhoA also has a role in MAPKi resistance, upstream of the focal adhesion kinase (FAK)-PI3K-AKT pathway^47^. To the best of our knowledge, RAC1 and RhoA have never been shown to regulate BRAF oncoprotein expression, which may be unique to ARF6.

ARF6-dependent survival may also help explain why tumor-specific deletion of *Arf6* significantly diminished tumor development and progression in BRAF^V600E^ PTEN^WT^ melanoma models^12^. While impaired tumor formation and sluggish growth were attributable to ARF6-dependent suppression of the adaptive immune response in that model^12^, our current findings suggest that ARF6 might also render tumor cells more resistant to apoptotic death incited by immune attack. More work is needed to understand ARF6-mediated tumor survival, including during immune-mediated tumor killing.

By interrogating ARF6 *in vitro* and in immunodeficient mice, we removed the influence of adaptive immunity and discovered an unanticipated role for ARF6 in tumor cell survival. While our findings support a mechanism whereby ARF6 activation fortifies BRAF^V600E^ protein synthesis, other ARF6 mechanisms may be at play. For example, we have previously shown that ARF6-GTP upregulated PI3K expression and AKT-signaling in melanoma while inhibition of ARF6 reduced PI3K and AKT activation^8^. In this current study, ARF6 was critical for tumor growth acceleration caused by loss of PTEN. Together these data support that ARF6 regulates the PI3K-AKT axis and as such, it is possible that ARF6 modulates PI3K-AKT driven anti-apoptotic signaling. Lastly, because ARF6 mediates internalization^55^ and recycling^56^ of integrins (i.e. focal adhesion turnover), ARF6 activity might be linked to FAK-dependent resistance to MAPK targeted therapy in melanoma^47^. Independent of these possibilities, our data reveal a previously unknown vulnerability in oncogenic BRAF signaling, ARF6, which may be exploitable for addressing DTP cell survival and targeted therapy resistance.

## METHODS

### Mouse husbandry, genotyping and RCAS virus delivery in vivo.

Animal studies were performed in accordance with a protocol approved by the University of Utah Institutional Animal Care and Use Committee (IACUC). Generation of the *Dct::TVA*; *Braf*^*V600E*^; *Cdkn2a*^*f/f*^, *Dct::TVA; Braf*^*V600E*^; *Cdkn2a*^*f/f*^; *Arf6*^*f/f*^, and *Dct::TVA; Braf*^*V600E*^; *Cdkn2a*^*f/f*^; *Pten*^*f/f*^ murine models have been described previously^8, 12^. The flank tumor incidence, onset, growth rate and overall survival were measured and calculated as described previously^12^. Both male and female animals were used in this study and were equally distributed across experimental groups. Prior analysis confirmed that sex does not influence tumor formation, tumor size, or survival onset in our model (PMID: 39098861, PMID: 33098202)

For the PDX cell line (MTG013) model, all animal studies were approved by the University of Utah IACUC and were performed in accordance with relevant guidelines and regulations by the Huntsman Cancer Institute (HCI) Preclinical Research Resource (PRR) laboratory. 10 females and 10 males of six to eight-week-old NOD rag gamma (NGR, NOD-*Rag1*^*null*^*IL2rg*^*null*^, NOD rag gamma, NOD-RG) mice, Jackson Laboratory stock 7799, were injected subcutaneously with 5 × 10^5^ cells in matrigel. Mice were treated with or without Dox chow (Envigo: Global 18% Protein Rodent Diet with 625ppm doxycycline. Cat# TD.01306.) five days after injection. Mice were monitored for health weekly, and tumor size was measured twice weekly using digital calipers; the tumor volume was calculated using the following formula: (length × width^2^/2).

### Cell lines

Authentication of all human melanoma cell lines were periodically confirmed by STR profiling in the University of Utah Genomics core facility using the Promega (Madison, WI) GenePrint 10 system, or by ATCC. A375, LOX-IMVI, UACC.62, were provided by Dr. M. VanBrocklin, HCI. A2058 cells were purchased from the ATCC (Cat# CRL11147D). SKMEL28 cells were provided by Dr. D. Grossman, HCI. A2058 and A375 were maintained in DMEM-high glucose (ThermoFisher Scientific, Cat# 11995073) supplemented with 10% v/v FBS (Atlas Biologicals, Cat# F-0500-DR), 1% v/v penicillin-streptomycin-glutamine (ThermoFisher Scientific, Cat# 10378016). LOX-IMVI, SKMEL28, and UACC.62 cells were maintained in PRMI1640-high glucose media (ThermoFisher Scientific, Cat# A1049101) supplemented with 10% v/v FBS, 1% v/v penicillin-streptomycin-glutamine.

Early passage, patient-derived MTG013/HCICM-013 and MTG030/HCI-CM030 melanoma cells were obtained from the HCI PRR laboratory. These primary cells were derived from tumor that was obtained from two distinct patients who provided written informed consent according to a tissue collection and usage protocols IRB 89989 and 10924, approved by the University of Utah Institutional Review Board. Access to these biospecimens is available through the HCI PRR lab. Patient-derived human melanoma cells were maintained in Mel2 media, which consists of 80% v/v MCDB 153 media (Sigma, Cat# M7403–10X1L), 20% v/v Leibovitz’s L-15 Media (ThermoFisher Scientific, Cat# 11415064), 2% v/v FBS, 1.68mM CaCI_2_, 1x Insulin-Transferrin-Selenium-Ethanolamine (ITS-X)(Fisher Scientific, Cat# 51500056), 5ng/mL EGF(Sigma, Cat# E-4127), 15ug/mL Bovine Pituitary Extract (ThermoFisher, Cat# 13028014), 1% v/v Penicillin-Streptomycin (ThermoFisher Scientific, Cat# 15070063).

Early passage murine tumor cell lines were derived from primary melanoma tumors induced in *Dct::TVA*; *Braf*^*V600E*^; *Cdkn2a*^*f/f*^ mices^8, 12^. Cell line 5588 = ARF6^WT^. Cell line 20000 = ARF6^NULL^. Cell line 6431 expresses ectopic ARF6^Q67L^. Cells were cultured with DMEM/ F12 HEPES (ThermoFisher Scientific, Cat # 37075) containing 10% v/v FBS, 1% v/v penicillin-streptomycin-glutamine, 1% v/v MEM Non-Essential Amino Acids Solution (ThermoFisher Scientific, Cat #11140050) under standard conditions at 37°C in a humidified atmosphere, 5% CO_2_. DF-1 and A375-TVA cells were provided by S. Holmen (HCI). DF-1 cells were maintained in DMEM-high glucose supplemented with 10% FBS, 0.5% v/v gentamicin (ThermoFisher Scientific, Cat# 15710072), and maintained at 39°C, with 5% CO2. A375-TVA cells were maintained in DMEM-high glucose supplemented with 10% FBS and 0.5% v/v gentamicin at 37°C with 5% CO2 and were used to verify RCAS/Cre expression in DF-1 cells.

Human colorectal carcinoma HT-29 cells were purchased from ATCC (Cat# HTB-38) and were maintained in ATCC-formulated McCoy’s 5a Medium Modified (ATCC, Cat# 30–2007), 10%v/v FBS, 1%v/v penicillin-streptomycin-glutamine. Human glioma DBTRG-05MG cells were purchased from ATCC (Cat# CRL-2020) and were maintained in ATCC-formulated RPMI-1640 Medium (Cat# 30–2001), 10%v/v FBS, 30mg/L L-proline (Sigma-Aldrich, Cat# 81709–10G), 35mg/L L-cystine (ThermoFisher Scientific, Cat# J63745.14), 3.57g/L HEPES (ThermoFisher Scientific, Cat# 15630080), 15mg/L hypoxanthine (Sigma-Aldrich, Cat# H9636–1G), 1mg/L adenosine triphosphate (Sigma-Aldrich, Cat# A6419–1G), 10mg/L adenine (Sigma-Aldrich, Cat# A2786–5G), 1mg/L thymidine (Sigma-Aldrich, Cat# T1895–1G), and 1%v/v penicillin-streptomycin-glutamine. Cells were incubated at 37°C in a humidified atmosphere with 5% CO_2_.

### RNA interference

Transient silencing of endogenous ARF6 was performed by sequential transfection of siRNA (*ARF6*, Qiagen Cat# 1027417; GeneGlobe S02757286), and compared to AllStars Negative Control siRNA (Qiagen, Cat# 1027181) at a final concentration of 40nM using Lipofectamine^™^ RNAiMAX transfection reagent (ThermoFisher Scientific, Cat# 13778150). Briefly, cells were seeded in a 6-well plate and first transfected with 40nM siRNA mixed with 7.5μL of Lipofectamine^™^ RNAiMAX transfection reagent. After 24 hours, transfections were repeated under the same conditions. Cells were collected 24 h after the second transfection for cell viability and western blot analyses.

For conditional *ARF6* silencing with short hairpin RNA (shRNA), MTG013 and MTG030 cells were stably transduced with a replication-incompetent retrovirus (piSMART-hEF1a-GFP-shARF6, see Key Resource Table) and cultured under 1μM puromycin selection. *In vitro*, stably transduced cell lines were treated with 1.0 μM doxycycline.

### Western blot and ARF6-GTP-pulldown

Cells were lysed using Pierce^®^ IP Lysis buffer (ThermoFisher Scientific, Cat # 87788) with 1X Halt^™^ Protease and Phosphatase Inhibitor Cocktail (ThermoFisher Scientific, Cat# 78442). Protein concentrations were determined using the Pierce^™^ BCA Protein Assay Kit (ThermoFisher Scientific, Cat# 23227). Cell lysates were boiled with SDS sample buffer. Proteins from the cell lysates were separated by SDS polyacrylamide gel electrophoresis (SDS–PAGE) and transferred to polyvinylidene difluoride (PVDF) membranes (ThermoFisher Scientific, Cat# 88518). The PVDF membranes were blocked with TBST (10 mM Tris-HCl, 150 mM NaCl, and 0.1% v/v Tween-20) containing 5% w/v skim milk and incubated with primary antibodies. After washing in TBST, membranes were incubated with HRPconjugated secondary antibodies and then washed with TBST before developing with Western Lightning^™^ Plus Chemiluminescence Reagent (PerkinElmer, Cat# NEL103001EA) or SuperSignal^™^ West Dura Extended Duration Substrate (ThermoFisher Scientific, Cat# 37075). Luminescent signal was detected using the Azure c300 or c600 (Azure Biosystems). ImageJ (NIH, Bethesda, MD, USA) was used to quantify the intensity of bands on the blots. Images were adjusted equally for brightness and contrast using ImageJ or Adobe Photoshop (Adobe Inc.).

ARF6-GTP pull-downs were performed using GGA3 PBD Agarose beads (Cell Biolabs, Cat# STA-419) as previously described^11^. Briefly, cells were treated with chemical compounds for the indicated time. After treatment, cells were lysed with pulldown lysis buffer (Cell Biolabs, Cat# 240102) including 1X Halt^™^ Protease and Phosphatase Inhibitor Cocktail. Lysates were centrifuged, supernatants were added to GGA3-conjugated beads and agitated for 1 hour at 4°C. Beads were washed in ARF6-pulldown lysis buffer and prepared for western blot analysis.

### Cell viability assay

Cell viability was detected by CellTiter-Glo^®^ Luminescent Cell Viability Assay (Promega, Cat# G7571). Briefly, 2000 cells/well were seeded in 96 well plates overnight. The next day, cell viability was measured before treatment (0-hour time point). After 48 or 72 hours of treatment, media were removed and replaced with the CellTiter-Glo^®^ Reagent. Luminescence was measured by Perkin Elmer EnVision Multi-Mode Plate Reader.

### Apoptosis Assay

Apoptosis was detected by RealTime Glo^™^ Annexin V Apoptosis Assay (Promega; Cat# JA1000). Briefly, 10,000 cells/well were seeded in 96 well plates overnight. The next day, cells were treated with serum starvation or chemical compounds to induce apoptosis plus apoptosis detection reagent. Annexin V luminescence was measured by Perkin Elmer EnVision Multi-Model Plate Reader.

### Drug tolerant colony formation

Human melanoma cells A375 were seeded at 10,000 cells per well in 6 well plates and treated with vemurafenib, dabrafenib, trametinib, and/or QS11 for 30 days. For colony formation assay with early passage, patient-derived MTG013 cells were seeded at 200,000 cells per well in 6 well plates and treated with dabrafenib, trametinib, and/or NAV-2729 for 14 days. For patient-derived MTG030 cells were treated with dabrafenib, trametinib, and/or doxycycline. Drugs were refreshed every 2–3 days. After 14 or 30 days of drug treatment, cells were fixed with methanol and stained with 0.5% crystal violet stains. Plates were scanned with LICOR Odyssey^®^ DLx scanner. Colony Area or intensity was measured by ImageJ^57^. Representative images were captured with a Nikon Automated Widefield Microscope.

### Cloning, viral transduction and generation of stable cell lines

The pTRIPZ lentviral system (used for cloning pTRIPZ-ARF6^WT^-V5 and pTRIPZ-ARF6^Q67L^-V5) was gifted from Dr. Todd W. Ridky^58^. ARF6^WT^-V5 and ARF6^Q67L^-V5 were inserted into the p-TRPIZ vector using the In-Fusion Snap Assembly system (Takarabio, Cat# 638945). HEK-293T cells were co-transfected with 2nd generation lentivirus packaging vectors (5μg pCMV-Gag/Pol, Addgene, Cat#35614; 1μg pCMV-VSVG, Addgene, Cat# 8454) and 5μg of expression constructs (including piSMART-hEF1a-TurboGFP-sh*ARF6*, see Key Resource Table) using Lipofectamine^™^ 3000 Transfection Reagent (Thermo Scientific, Cat# L3000008). Viral supernatants were harvested 48 hours and 72 hours post-transfection and filtered through a 0.45 μm filter. Filtered viral supernatants were applied to target cell lines together with 10 μg/ml of Polybrene (Sigma Cat# TR-1003). After infection, cells were placed in fresh media for three days before selection with 1μM puromycin for 14 days. Doxycycline dose response treatments confirmed ectopic expression or knockdown efficiency. Stable cell lines were maintained in 1μm puromycin.

Adenoviral ARF6^Q67L^ was created by Vector Biolabs as previously described^11^. Cells were infected with 10^7^ pfu/mL virus and incubated for 24 hours prior to experimentation.

### Proteomics

Protein extraction and reverse-phase protein array of frozen mouse tumors were performed by the MD Anderson Cancer Center Functional Proteomic RPPA Core Facility.

### Quantitative reverse transcription polymerase chain reaction (qRT-PCR)

Total RNA was isolated from A375 cells after doxycycline-induced expression of ARF6^Q67L^. Cells were untreated or treated with 1μM doxycycline for 4, 8, 24, or 48 hours, then collected and stored in RNAlater (ThermoFisher Scientific, Cat# AM7024). RNA was extracted using RNeasy Plus kit (Qiagen, Cat# 74034) according to manufacturer's instructions. Extracted RNA from each sample was converted into cDNA using SuperScript IV VILO (SSIV VILO) Master Mix (ThermoFisher Scientific, Cat# 11756050). qRT-PCR was performed in triplicate for each sample using PowerUp^™^ SYBR^™^ Green Master Mix (ThermoFisher Scientific, Cat# A25780) on the QuantStudio^™^ 6 Flex Real-Time PCR System (ThermoFisher Scientific) in 96-well plates. Primers used for qRT-PCR are shown in the Key Resource Table. The specificity of the amplicons was assessed by melting curve analyses. Relative mRNA expression of each gene was calculated using the number of cycles needed to reach the crossing threshold of detection (CT) and normalized to the expression of *GAPDH*.

### Immunofluorescence

Murine tumors were embedded and frozen in Tissue-Tek^®^ O.C.T. compound. Tissues were sectioned 6–10μm thick using a cryostat. The tissue was fixed to the slides with acetone followed by three rinses in “PBSA” (1× PBS + 0.1% sodium azide). Slides were permeabilized with 1% bovine serum albumin (BSA) + 0.1% Saponin solution, followed by blocking in PBSA + 3% v/v BSA for 60 minutes. After blocking, the slides were incubated with the primary antibody overnight at 4°C. The next day, the slides were washed with PBSA and incubated with secondary antibody for 1 hour at room temperature, then washed again with PBSA After washing the slides before counterstaining with DAPI for 30 minutes at room temperature, followed by a 5-minute wash in PBSA, and mounting in 40% w/v polyvinyl pyrrolidone + 4% v/v glycerol + 0.1% sodium azide dissolved in 1 mol/L Tris, pH 8.0. Images were collected on an Olympus Fluoview1000 scanning laser confocal microscope at 1,200x magnification. Quantification of fluorescent signals on mouse tumor tissue was performed in ImageJ. Final signal intensity for BRAF, pMEK and pERK was calculated by total green signal count divided by the number of nuclei (DAPI stained).

### Statistical Analysis

Details of each statistical analysis are included in the figure legends. Statistical tests were performed using Prism software (GraphPad). Quantitative values are shown with or represented as the mean of at least three biologic replicates.

## Supplementary Material

This is a list of supplementary files associated with this preprint. Click to download.

- FigS1071425.tif
- FigS2070925.tif
- FigS3070925.tif
- KeyResourcetable1.docx

## Data Availability

The data generated herein are available from the corresponding author upon request.

## References

[1] Hanahan D. Hallmarks of Cancer: New Dimensions. Cancer Discov 12, 31–46 (2022). 35022204 10.1158/2159-8290.CD-21-1059

[2] Pu Y. Drug-tolerant persister cells in cancer: the cutting edges and future directions. Nat Rev Clin Oncol 20, 799–813 (2023). 37749382 10.1038/s41571-023-00815-5

[3] He J. Drug tolerant persister cell plasticity in cancer: A revolutionary strategy for more effective anticancer therapies. Signal Transduct Target Ther 9, 209 (2024). 39138145 10.1038/s41392-024-01891-4 PMC11322379

[4] Liu S., Jiang A., Tang F., Duan M. & Li B. Drug-induced tolerant persisters in tumor: mechanism, vulnerability and perspective implication for clinical treatment. Mol Cancer 24, 150 (2025). 40413503 10.1186/s12943-025-02323-9 PMC12102949

[5] Lavoie H., Gagnon J. & Therrien M. ERK signalling: a master regulator of cell behaviour, life and fate. Nat Rev Mol Cell Biol 21, 607–632 (2020). 32576977 10.1038/s41580-020-0255-7

[6] Cancer Genome Atlas N. Genomic Classification of Cutaneous Melanoma. Cell 161, 1681–1696 (2015). 26091043 10.1016/j.cell.2015.05.044 PMC4580370

[7] Hanrahan A.J., Chen Z., Rosen N. & Solit D.B. BRAF - a tumour-agnostic drug target with lineage-specific dependencies. Nat Rev Clin Oncol 21, 224–247 (2024). 38278874 10.1038/s41571-023-00852-0 PMC11857949

[8] Yoo J.H. The Small GTPase ARF6 Activates PI3K in Melanoma to Induce a Prometastatic State. Cancer Res 79, 2892–2908 (2019). 31048499 10.1158/0008-5472.CAN-18-3026 PMC7197377

[9] Tague S.E., Muralidharan V. & D'Souza-Schorey C. ADP-ribosylation factor 6 regulates tumor cell invasion through the activation of the MEK/ERK signaling pathway. Proc Natl Acad Sci U S A 101, 9671–9676 (2004). 15210957 10.1073/pnas.0403531101 PMC470733

[10] Richards J.R. Activation of NFAT by HGF and IGF-1 via ARF6 and its effector ASAP1 promotes uveal melanoma metastasis. Oncogene 42, 2629–2640 (2023). 37500798 10.1038/s41388-023-02792-6 PMC11008337

[11] Grossmann A.H. The small GTPase ARF6 stimulates beta-catenin transcriptional activity during WNT5A-mediated melanoma invasion and metastasis. Sci Signal 6, ra14 (2013). 23462101 10.1126/scisignal.2003398 PMC3961043

[12] Wee Y. Tumour-intrinsic endomembrane trafficking by ARF6 shapes an immunosuppressive microenvironment that drives melanomagenesis and response to checkpoint blockade therapy. Nat Commun 15, 6613 (2024). 39098861 10.1038/s41467-024-50881-1 PMC11298541

[13] Yoo J.H. ARF6 Is an Actionable Node that Orchestrates Oncogenic GNAQ Signaling in Uveal Melanoma. Cancer Cell 29, 889–904 (2016). 27265506 10.1016/j.ccell.2016.04.015 PMC5027844

[14] Straussman R. Tumour micro-environment elicits innate resistance to RAF inhibitors through HGF secretion. Nature 487, 500–504 (2012). 22763439 10.1038/nature11183 PMC3711467

[15] Anastas J.N. WNT5A enhances resistance of melanoma cells to targeted BRAF inhibitors. J Clin Invest 124, 2877–2890 (2014). 24865425 10.1172/JCI70156 PMC4071371

[16] O'Connell M.P. Hypoxia induces phenotypic plasticity and therapy resistance in melanoma via the tyrosine kinase receptors ROR1 and ROR2. Cancer Discov 3, 1378–1393 (2013). 24104062 10.1158/2159-8290.CD-13-0005 PMC3918498

[17] Behera R. Inhibition of Age-Related Therapy Resistance in Melanoma by Rosiglitazone-Mediated Induction of Klotho. Clin Cancer Res 23, 3181–3190 (2017). 28232477 10.1158/1078-0432.CCR-17-0201 PMC5474161

[18] D'Souza-Schorey C. & Chavrier P. ARF proteins: roles in membrane traffic and beyond. Nat Rev Mol Cell Biol 7, 347–358 (2006). 16633337 10.1038/nrm1910

[19] Brooks R., Williamson R. & Bass M. Syndecan-4 independently regulates multiple small GTPases to promote fibroblast migration during wound healing. Small GTPases 3, 73–79 (2012). 22790193 10.4161/sgtp.19301 PMC3408980

[20] Tsai M.T. Regulation of HGF-induced hepatocyte proliferation by the small GTPase Arf6 through the PIP2-producing enzyme PIP5K1A. Sci Rep 7, 9438 (2017). 28842595 10.1038/s41598-017-09633-z PMC5572707

[21] Nieuwenhuis B. & Eva R. ARF6 and Rab11 as intrinsic regulators of axon regeneration. Small GTPases 11, 392–401 (2020). 29772958 10.1080/21541248.2018.1457914 PMC6124649

[22] Van Acker T. The small GTPase Arf6 is essential for the Tram/Trif pathway in TLR4 signaling. J Biol Chem 289, 1364–1376 (2014). 24297182 10.1074/jbc.M113.499194 PMC3894321

[23] Montealegre S. & van Endert P.M. Endocytic Recycling of MHC Class I Molecules in Non-professional Antigen Presenting and Dendritic Cells. Front Immunol 9, 3098 (2018). 30666258 10.3389/fimmu.2018.03098 PMC6330327

[24] Marquer C. Arf6 controls retromer traffic and intracellular cholesterol distribution via a phosphoinositide-based mechanism. Nat Commun 7, 11919 (2016). 27336679 10.1038/ncomms11919 PMC4931008

[25] Gogulamudi V.R. Heterozygosity for ADP-ribosylation factor 6 suppresses the burden and severity of atherosclerosis. PLoS One 18, e0285253 (2023). 37163513 10.1371/journal.pone.0285253 PMC10171652

[26] Hongu T. Arf6 regulates tumour angiogenesis and growth through HGF-induced endothelial beta1 integrin recycling. Nat Commun 6, 7925 (2015). 26239146 10.1038/ncomms8925

[27] Zhu W. Interleukin receptor activates a MYD88-ARNO-ARF6 cascade to disrupt vascular stability. Nature 492, 252–255 (2012). 23143332 10.1038/nature11603 PMC3521847

[28] Jones C.A. Slit2-Robo4 signalling promotes vascular stability by blocking Arf6 activity. Nat Cell Biol 11, 1325–1331 (2009). 19855388 10.1038/ncb1976 PMC2854659

[29] Zhu W. Small GTPase ARF6 controls VEGFR2 trafficking and signaling in diabetic retinopathy. J Clin Invest (2017). 10.1172/JCI91770 PMC5707163 29058688

[30] Davis C.T. ARF6 inhibition stabilizes the vasculature and enhances survival during endotoxic shock. J Immunol 192, 6045–6052 (2014). 24835390 10.4049/jimmunol.1400309 PMC4291019

[31] Zhang Q. Small-molecule synergist of the Wnt/beta-catenin signaling pathway. Proc Natl Acad Sci U S A 104, 7444–7448 (2007). 17460038 10.1073/pnas.0702136104 PMC1863490

[32] da Rocha Dias S. Activated B-RAF is an Hsp90 client protein that is targeted by the anticancer drug 17-allylamino-17-demethoxygeldanamycin. Cancer Res 65, 10686–10691 (2005). 16322212 10.1158/0008-5472.CAN-05-2632

[33] Grbovic O.M. V600E B-Raf requires the Hsp90 chaperone for stability and is degraded in response to Hsp90 inhibitors. Proc Natl Acad Sci U S A 103, 57–62 (2006). 16371460 10.1073/pnas.0609973103 PMC1325013

[34] D'Souza-Schorey C. ARF6 targets recycling vesicles to the plasma membrane: insights from an ultrastructural investigation. J Cell Biol 140, 603–616 (1998). 9456320 10.1083/jcb.140.3.603 PMC2140168

[35] Prigent M. ARF6 controls post-endocytic recycling through its downstream exocyst complex effector. J Cell Biol 163, 1111–1121 (2003). 14662749 10.1083/jcb.200305029 PMC2173613

[36] Hafner M. Inhibition of cytohesins by SecinH3 leads to hepatic insulin resistance. Nature 444, 941–944 (2006). 17167487 10.1038/nature05415

[37] Kawakami H. Mutant BRAF Upregulates MCL-1 to Confer Apoptosis Resistance that Is Reversed by MCL-1 Antagonism and Cobimetinib in Colorectal Cancer. Mol Cancer Ther 15, 3015–3027 (2016). 27765849 10.1158/1535-7163.MCT-16-0017 PMC5136313

[38] Yang J.Y. ERK promotes tumorigenesis by inhibiting FOXO3a via MDM2-mediated degradation. Nat Cell Biol 10, 138–148 (2008). 18204439 10.1038/ncb1676 PMC2376808

[39] He Y. Targeting PI3K/Akt signal transduction for cancer therapy. Signal Transduct Target Ther 6, 425 (2021). 34916492 10.1038/s41392-021-00828-5 PMC8677728

[40] Bok I. A Versatile ES Cell-Based Melanoma Mouse Modeling Platform. Cancer Res 80, 912–921 (2020). 31744817 10.1158/0008-5472.CAN-19-2924 PMC7024666

[41] Xu X. PTEN Lipid Phosphatase Activity Suppresses Melanoma Formation by Opposing an AKT/mTOR/FRA1 Signaling Axis. Cancer Res 84, 388–404 (2024). 38193852 10.1158/0008-5472.CAN-23-1730 PMC10842853

[42] Cho J.H. AKT1 Activation Promotes Development of Melanoma Metastases. Cell Rep 13, 898–905 (2015). 26565903 10.1016/j.celrep.2015.09.057 PMC4646731

[43] Yaeger R. A Next-Generation BRAF Inhibitor Overcomes Resistance to BRAF Inhibition in Patients with BRAF-Mutant Cancers Using Pharmacokinetics-Informed Dose Escalation. Cancer Discov 14, 1599–1611 (2024). 38691346 10.1158/2159-8290.CD-24-0024 PMC11372368

[44] in NCCN Clinical Practice Guidelines in Oncology, Edn. V2.2025 (National Comprehensive Cancer Network, 2025).

[45] Sondka Z. COSMIC: a curated database of somatic variants and clinical data for cancer. Nucleic Acids Res 52, D1210–D1217 (2024). 38183204 10.1093/nar/gkad986 PMC10767972

[46] Smith E.A. Receptor tyrosine kinase inhibition leads to regression of acral melanoma by targeting the tumor microenvironment. J Exp Clin Cancer Res 43, 317 (2024). 39627834 10.1186/s13046-024-03234-1 PMC11613472

[47] Lubrano S. FAK inhibition combined with the RAF-MEK clamp avutometinib overcomes resistance to targeted and immune therapies in BRAF V600E melanoma. Cancer Cell 43, 428–445 e426 (2025). 40020669 10.1016/j.ccell.2025.02.001 PMC11903146

[48] Lito P. Relief of profound feedback inhibition of mitogenic signaling by RAF inhibitors attenuates their activity in BRAFV600E melanomas. Cancer Cell 22, 668–682 (2012). 23153539 10.1016/j.ccr.2012.10.009 PMC3713778

[49] Polier S. ATP-competitive inhibitors block protein kinase recruitment to the Hsp90-Cdc37 system. Nat Chem Biol 9, 307–312 (2013). 23502424 10.1038/nchembio.1212 PMC5695660

[50] Jia X. Protein translation: biological processes and therapeutic strategies for human diseases. Signal Transduct Target Ther 9, 44 (2024). 38388452 10.1038/s41392-024-01749-9 PMC10884018

[51] Livingston N.M. Bursting translation on single mRNAs in live cells. Mol Cell 83, 2276–2289 e2211 (2023). 37329884 10.1016/j.molcel.2023.05.019 PMC10330622

[52] Gadal S. Tumorigenesis Driven by BRAFV600E Requires Secondary Mutations that Overcome it's Feedback Inhibition of RAC1 and Migration. Cancer Res (2025). 10.1158/0008-5472.CAN-24-2220 PMC12046322 39992718

[53] Lionarons D.A. RAC1(P29S) Induces a Mesenchymal Phenotypic Switch via Serum Response Factor to Promote Melanoma Development and Therapy Resistance. Cancer Cell 36, 68–83 e69 (2019). 31257073 10.1016/j.ccell.2019.05.015 PMC6617390

[54] Watson I.R. The RAC1 P29S hotspot mutation in melanoma confers resistance to pharmacological inhibition of RAF. Cancer Res 74, 4845–4852 (2014). 25056119 10.1158/0008-5472.CAN-14-1232-T PMC4167745

[55] Yue J. Microtubules regulate focal adhesion dynamics through MAP4K4. Dev Cell 31, 572–585 (2014). 25490267 10.1016/j.devcel.2014.10.025 PMC4261153

[56] Ghosh M. CD13 tethers the IQGAP1-ARF6-EFA6 complex to the plasma membrane to promote ARF6 activation, beta1 integrin recycling, and cell migration. Sci Signal 12 (2019). 10.1126/scisignal.aav5938 PMC6756473 31040262

[57] Guzman C., Bagga M., Kaur A., Westermarck J. & Abankwa D. ColonyArea: an ImageJ plugin to automatically quantify colony formation in clonogenic assays. PLoS One 9, e92444 (2014). 24647355 10.1371/journal.pone.0092444 PMC3960247

[58] McNeal A.S. CDKN2B Loss Promotes Progression from Benign Melanocytic Nevus to Melanoma. Cancer Discov 5, 1072–1085 (2015). 26183406 10.1158/2159-8290.CD-15-0196 PMC4592422

